# Increased gulf stream warm core ring formations contributes to an observed increase in salinity maximum intrusions on the Northeast Shelf

**DOI:** 10.1038/s41598-023-34494-0

**Published:** 2023-05-09

**Authors:** Adrienne Silver, Avijit Gangopadhyay, Glen Gawarkiewicz, Paula Fratantoni, Jenifer Clark

**Affiliations:** 1 School for Marine Science and Technology, University of Massachusetts Dartmouth, 836 S Rodney French Blvd, New Bedford, MA 02744 USA; 2 Physical Oceanography Department, Woods Hole Oceanographic Institution, 266 Woods Hole Road, Woods Hole, MA 02543 USA; 3 Northeast Fisheries Science Center, NOAA NMFS, 166 Water Street, Woods Hole, MA 02543 USA; 4 Jenifer Clark’s Gulfstream, 3160 Lacrosse Court, Dunkirk, MD 20754 USA

**Keywords:** Physical oceanography, Environmental impact, Climate change

## Abstract

We present observational evidence of a significant increase in Salinity Maximum intrusions in the Northeast US Shelf waters in the years following 2000. This increase is subsequent to and influenced by a previously observed regime-shift in the annual formation rate for Gulf Stream Warm Core Rings, which are relatively more saline than the shelf waters. Specifically, mid-depth salinity maximum intrusions, a cross-shelf exchange process, has shown a quadrupling in frequency on the shelf after the year 2000. This increase in intrusion frequency can be linked to a similar increase in Warm Core Ring occupancy footprint along the offshore edge of the shelf-break which has greatly increased the abundance of warm salty water within the Slope Sea. The increased ring occupancy footprint along the shelf follows from the near doubling in annual Warm Core Ring formation rate from the Gulf Stream. The increased occurrence of intrusions is likely driven by a combination of a larger number of rings in the slope sea and the northward shift in the GS position which may lead to more interactions between rings and the shelf topography. These results have significant implications for interpreting temporal changes in the shelf ecosystem from the standpoint of both larval recruitment as well as habitability for various important commercial species.

## Introduction

**Figure 1:**
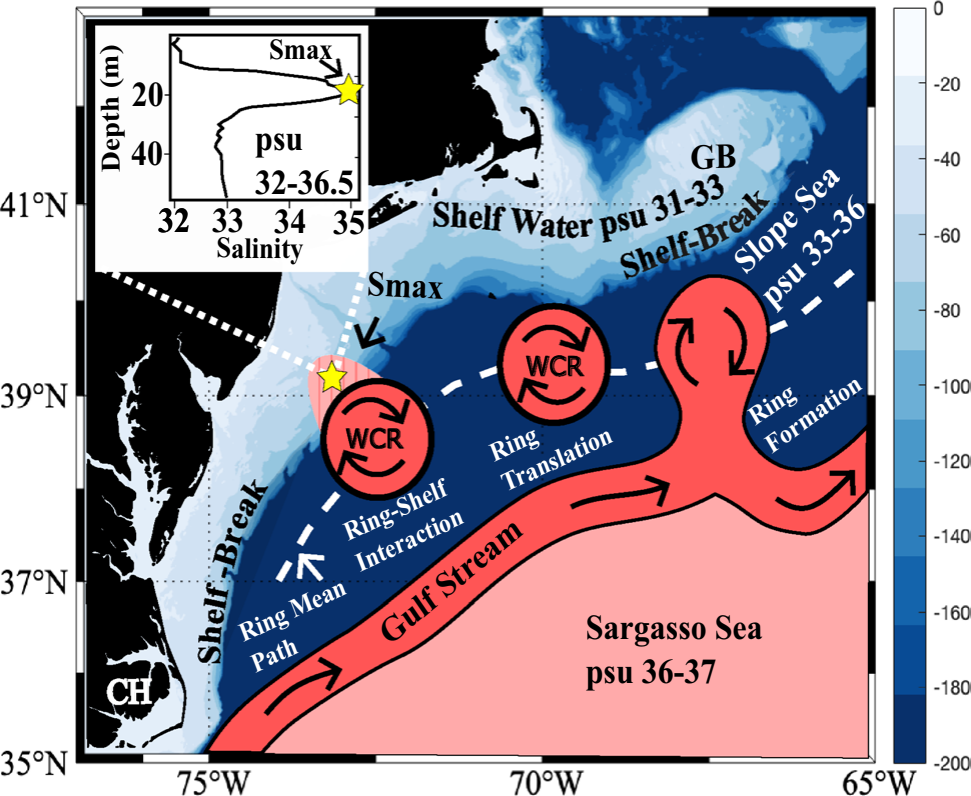
Schematic illustrating the proposed hypothesis that offshore forcing by the Gulf Stream drives the observed increase in the frequency of intrusions on the Northeast US Shelf. This increasing rate of intrusions starts with the doubling in the annual formation rate of Gulf Stream Warm Core Rings (Gangopadhyay et al., 2019). These rings then translate through the Slope Sea along the ring mean path (shown by the white dashed line) until they encounter the shelf between Georges Bank (labeled GB) and Cape Hatteras (labeled CH)^13^. The salty water within the Warm Core Rings interacts with the shelfbreak to form a Salinity Maximum intrusion. The top left corner of the schematic shows an example vertical salinity profile containing a salinity maximum intrusion from the EcoMon data. The yellow star marks the maximum salinity. This image was generated using Inkscape^14^ and M_Map^15^.

There have been a number of changes observed in the large-scale circulation of the North Atlantic in recent years. For example, the Atlantic Meridional Overturning circulation has been observed to be slowing^1^, the Gulf Stream has become more unstable^2^, and the number of Warm Core Rings formed from the Gulf Stream has roughly doubled since 2000^3^. There have also been notable changes on the Northeast US Shelf (Cape Hatteras to Cape Cod) with increased marine heatwaves^4,5^, biological shifts^6–8^ and increased shelfbreak exchange events^9^. One such shelfbreak exchange process is the mid-depth salinity maximum intrusion in which warm salty water from the continental Slope intrudes onto the continental shelf^10^. The schematic in Fig. 1 presents the geographical set up of this slope-shelf system with large-scale Gulf Stream Current, mesoscale Warm Core Rings, and shelf-scale salinity intrusions. An example profile of a salinity maximum intrusion is shown in the top left corner of Fig. 1.

**Figure 2:**
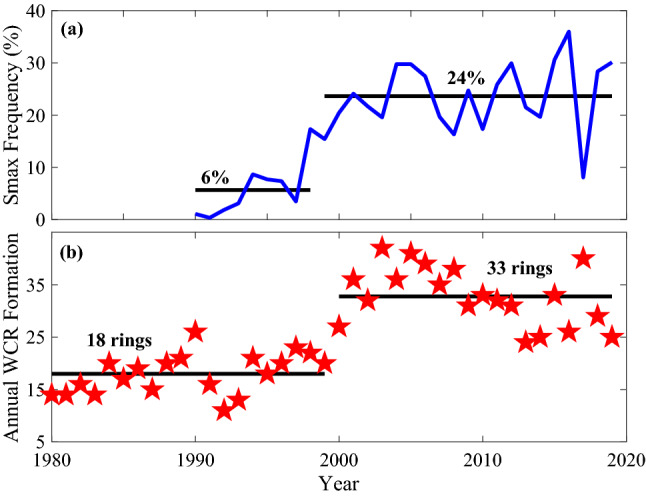
(**a**) Salinity maximum intrusion frequency showing the quadrupling increase in recent decades. The black line indicates a possible regime shift around 1998. (**b**) Annual Warm Core Ring formation numbers shows the significant regime shift in 2000.

Lentz^10^ conducted a census of salinity maximum intrusions using an archive of hydrographic profiles observed on the continental shelf between Cape Hatteras and the northeastern tip of Georges Bank between 1960 and 1998. Within this census, salinity maximum intrusions were present in 11% of the profiles examined. An updated census by Gawarkiewicz et al^11^, using both data from the Ecosystem Monitoring Program (EcoMon) and the Commercial Fisheries Research Foundation/Woods Hole Oceanographic Institution Shelf Research Fleet, found that the number of salinity maximum intrusions has greatly increased, occurring over 20% of the time in recent years, with intrusions also having a larger salinity anomaly and extending further inshore. It was noted that the change from a lower to a higher high salinity maximum intrusion frequency appeared to occur around the year 2000, the same year that an observed regime shift occurred in the number of annual Warm Core Ring formations^3^ (Fig. 2b). Before 2000, on average the Gulf Stream produced 18 rings per year. After 2000, this number nearly doubled, with an average of 33 rings forming per year.

Flagg et al.^12^ noted that high salinity water must be present over the upper continental slope adjacent to the shelfbreak in order for salinity maximum intrusions to occur. The doubling in the number of Warm Core Rings formed by the Gulf Stream reported by Gangopadhyay et al.^3^ indicates that the Slope Sea could be receiving double the amount of warm salty water, making conditions more favorable for salinity maximum intrusions to occur. We propose that the regime shift observed in the Gulf Stream system has led to increasing occurrences of Salinity Maximum intrusions on the Northeast US Shelf due to the larger footprint of warm rings in the Slope Sea (Fig. 1). This implies a substantial increase in the exchange of waters between the continental shelf and slope in this region.

## Results

### Interannual and seasonal variability of warm core rings and shelf intrusions

Following Gawarkiewicz et al.^11^, we used the EcoMon data (1990–2019) to assess the interannual variability of the frequency of salinity maximum intrusions relative to the presence of Gulf Stream Warm Core Rings. See Methods for data description. The salinity maximum intrusion frequency for a given year is calculated as the percentage of the total number of EcoMon profiles which contain intrusions for that year. Only profiles sampled within 50 km of the 100 m isobath were considered in the frequency calculation so that the relationship between Warm Core Rings and intrusions could be compared more directly and the shoreward advection timescale of the intrusions could be ignored as a first approximation. In addition, no profiles were considered south of 38°N in order to eliminate salinity maximum intrusions which are generated via direct Gulf Stream influence near Cape Hatteras.

Similar to Gawarkiewicz et al.^11^ (their Figure 13) there appears to be a large increase in the salinity maximum intrusion frequency around the year 2000 (Fig. 2a). To test whether this increase can be considered as a significant regime shift, we followed the methodology of Gangopadhyay et al.^3^ using several different shift detection methods (see ‘Methods’ section).

Interestingly, most of the shift-detection analyses for the Salinity Maximum Intrusion frequency show a statistically significant regime shift in 2000 for the longer cutoff lengths (15–20) and a shift in 1998 for the shorter cutoff lengths (5 and 10) both of which are around the time that have been previously recognized as an observed regime shift in Warm Core Ring formation^3^. In addition, the MATLAB changepoint algorithm also found a significant shift in 1998 matching with the STARS results at shorter cutoff lengths. The RSS for either regime shift occurring in 1998 or 2000 is also lower than when the data is fit with a linear trend (983, 1110, 1390 respectively). We recognize that the length of the first regime in the available ECOMON dataset (1990–1999) is short; thus one could interpret this change in salinity maximum intrusions as an “increasing trend” in the recent decades rather than a pure “regime shift”. However the intrusion analysis that we did with the EcoMon data only considered the part of the record when CTD’s were used for sampling (beginning in 1990). Lentz^10^ considered the full hydrographic record spanning 1960–1998 and found an overall frequency of 11%. This frequency cannot be directly compared to ours due to the subset of profiles that we used restricting to 50 km onshore of the shelfbreak. When we use the full EcoMon domain matching Lentz^10^ we find a similar shift with an increase from 5% before 2000 to 19% after 2000 with the 11% in the study by Lentz 2003 being around half of what we see in the recent time period. Additionally, Gawarkiewicz et al.^11^ used a higher threshold criterion (difference between the maximum salinity within an intrusion and the ambient salinity, ∆S) for identifying an intrusion than Lentz^10^, implying that the 11% found in Lentz^10^ (∆S = 0.1 psu) would be even lower following the Gawarkiewicz et al.^11^ methodology (∆S = 0.2 psu). This indicates that the low intrusion frequency period before 2000 extends back at least 3 more decades and that the observed shift, with a quadrupling of frequency in the salinity maximum intrusion, is significant.

Several metrics quantifying the impact of Warm Core Rings hitting the shelf were also calculated. The first was an along shelf Warm Core Ring occupancy footprint index (RFI) which is a space–time weighted metric of ring occupancy (more details provided in the Methods section). In addition, a similar metric was calculated measuring the length of time in days per year that rings were present along the shelfbreak after hitting the shelf to estimate the impact in time. Finally, the last two metrics to be examined were the number of rings that hit the shelfbreak per year and the number of rings that demised along the shelfbreak per year. All of these ring-related metrics show an increasing trend over the 40-year period with a subtle shift around the year 2000 (see Figure S1).

Harden et al.^16^ report that stratification in the shelf waters during the summer has been increasing in recent years (2003–2013). Increased stratification along with the stratified season starting and ending later in a year could set up the shelf waters to be more susceptible to the formation of intrusions. This process, along with the increased occupancy of Warm Core Rings along the shelfbreak likely contributes to the large increase in the frequency of Salinity Maximum Intrusions.

**Figure 3:**
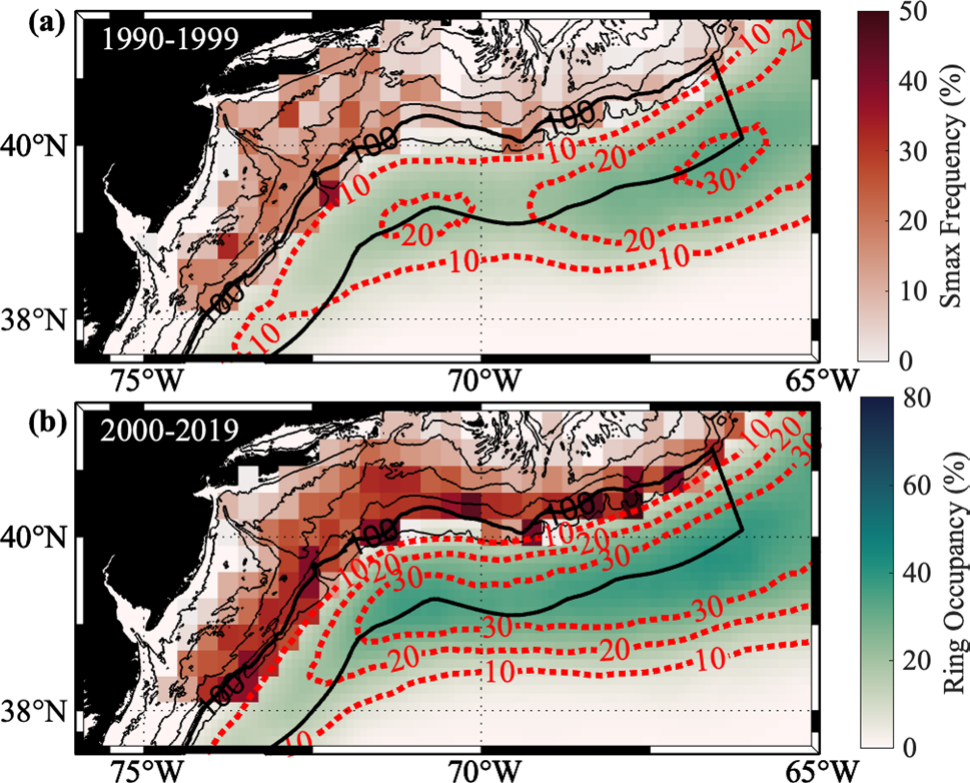
Red shading over the shelf shows the Salinity Maximum Intrusion frequency within 0.3 by 0.3 degree bins from EcoMon Data during (**a**) Regime 1 (1990–1999) and (**b**) Regime 2 (2000–2019). The green–blue shading within the Slope Sea shows the percent of the year each region was occupied by Warm Core Rings with the 10, 20, and 30% occupancy lines plotted in red dotted lines. Thick black outline shows the region within which the ring footprint index is calculated. This region is bounded by the 100 m isobath along its inshore edge and extends 1-degree offshore. The map in this figure was generated using M_Map^15^.

Furthermore, the spatial distribution of the Salinity Maximum Intrusion frequency and the Warm Core Ring occupancy during the 1990s and for 2000–2019 (Fig. 3) shows that this increase in magnitude spans the entire shelfbreak. After the Ring formation regime shift in 2000, the frequency of Salinity Maximum Intrusions is much higher along the 100 m isobath (marked by the northern edge of the black box region) and further inshore. Additionally, the ring occupancy increases along the full extent of the shelfbreak, matching the pattern of the Salinity Maximum Intrusion frequency. At a monthly time scale, one can see clustering of profiles with Salinity Maximum Intrusions around locations of high ring occupancy (See the monthly animation for a low and high year of intrusion/ring occupancy in Figure S2 as well the monthly figures found at https://doi.org/10.5281/zenodo.7859078).

**Figure 4:**
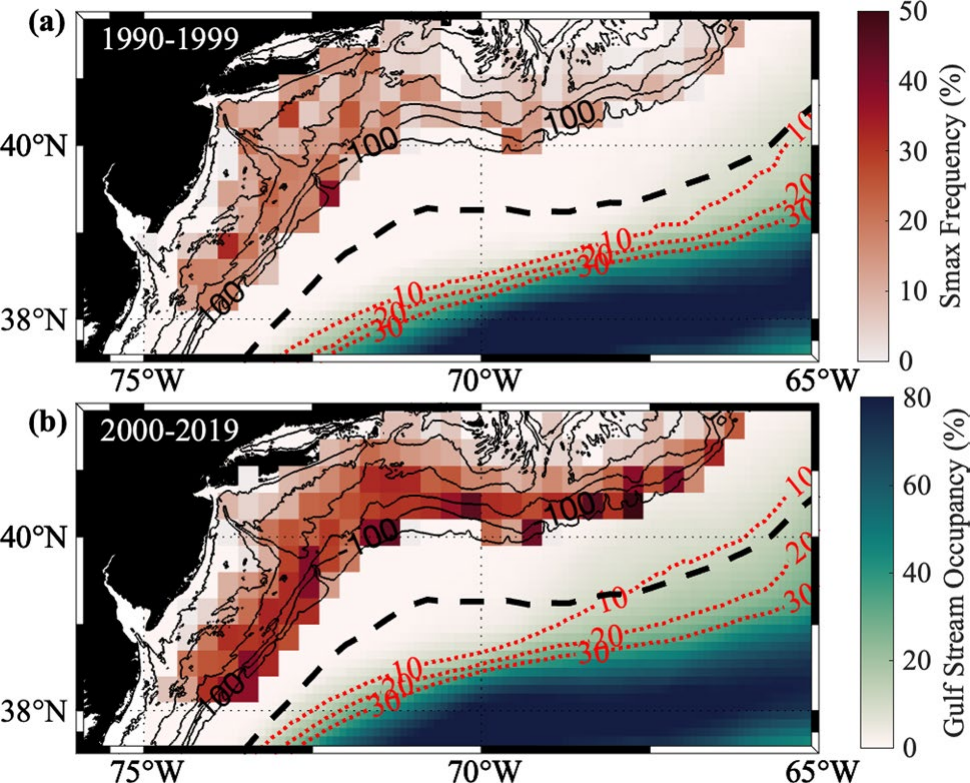
Red shading over the shelf shows the Salinity Maximum Intrusion frequency within 0.3 by 0.3 degree bins from EcoMon Data during (**a**) Period 1 (1990–1999) and (**b**) Period 2 (2000–2019). This matches with what is seen in Fig. 3. The green–blue shading within the Slope Sea shows the percent of the year each region was occupied by the Gulf Stream with the 10, 20, and 30% occupancy lines plotted in red dotted lines. The dashed black line shows the mean path of Warm Core Rings taken from the Silver et al.^18^ study. The map in this figure was generated using M_Map^15^.

Another possible driver of the increase in salinity maximum intrusions could be changes within the Gulf Stream itself. Andres^2^ reported that the destabilization point of the Gulf Stream moved westward in the late 1990s. Additionally, Wang et al.^17^ report that the Gulf Stream has moved northward west of the New England Seamount Chain (~ 65°W) in recent years. Both larger meanders in the Stream occurring further to the west and a northward bias in the current’s axis could lead to more interactions between the Gulf Stream and the shelfbreak and subsequently more Salinity Maximum intrusions. To examine the influence of the Gulf Stream we looked at the Gulf Stream Occupancy using the 25 cm isoheight (representing the Gulf Stream axis) from Sea Surface Height absolute dynamic topography (Fig. 4). The Gulf Stream was then assumed to span a half a degree north and south of the 25 cm isoheight contour. From this the percent of time the Gulf Stream was present in 0.1 by 0.1 degree bins in the Slope Sea was calculated. The Gulf Stream occupancy from before and after 2000 (Fig. 4a,b respectively) shows that there does appear to be a slight northward movement in the Stream’s position during this time frame. However, the Gulf Stream remains far enough offshore across most of the region so that direct interactions between the Stream and the shelfbreak are unlikely. The one region where the Gulf Stream comes fairly close to the shelfbreak is southeast of Georges Bank, which is east of the NE shelf region. The increase in Gulf Stream activity in this region could lead to more salinity maximum intrusions. Comparing Figs. 3 and 4 however shows that this region is much more likely to be occupied by a Warm Core Ring than by the Gulf Stream, indicating that Warm Core Rings are likely still the driving cause of the increase in Salinity Maximum Intrusions even to the east of the NE Shelf.

**Figure 5:**
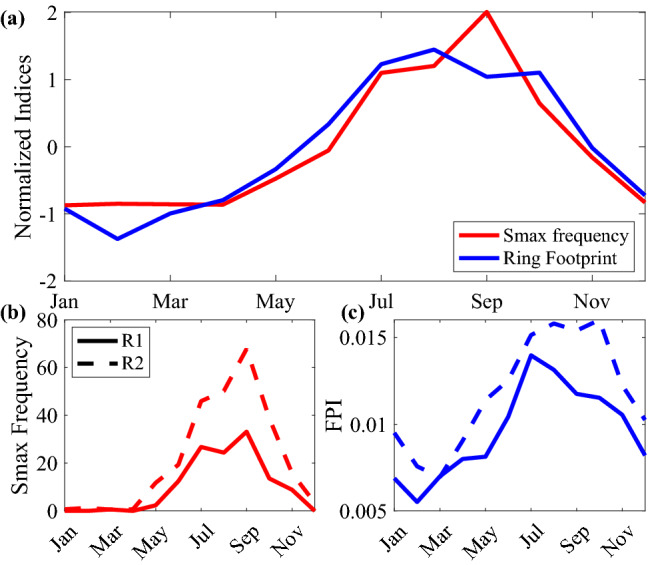
(**a**) Similarity of Seasonal pattern between the salinity maximum intrusion frequency and ring footprint index. (**b**) Salinity maximum intrusion frequency before (solid line) and after (dashed line) the Ring formation regime shift in 2000. (**c**) Ring footprint index before (solid line) and after (dashed line) the Ring formation regime shift in 2000.

Salinity maximum intrusion frequency and RFI are also correlated on a seasonal time scale (Fig. 5a). Both show a sustained peak from July to October and sustained valley from December to April. The annual cycles of the two seasonal indices are strikingly similar. The seasonal averages of salinity maximum intrusion frequency and RFI before and after the Ring formation regime shift show very similar patterns with just a change in amplitude after the Ring formation shift (Fig. 5b,c).

### Salinity maximum intrusions and warm core ring association

To further assess the possible relationship between Salinity Maximum Intrusions and Warm Core Rings, each EcoMon profile with a known Salinity Maximum Intrusion was compared to the proximity of nearby Warm Core Rings. Like the method used to calculate Salinity Maximum Intrusion Frequency, EcoMon profiles were only considered within 50 km of the 100 m isobath. For each profile a region of proposed ring influence was then drawn along the shelfbreak. This region of ring influence has a western edge at the location of the intrusion and extends 100 km east along the 100 m isobath and half a degree into the Slope Sea. The region was drawn to the east due to the average southwest transport on the shelf. This region of ring influence therefore accounts for rings situated directly south of the intrusion as well as those that are directly to the east. The presence of Warm Core Rings within this region were assessed for one month prior to the intrusion sighting. It was found that 72.3% of Salinity Maximum Intrusions observed over the study period (1990–2019) are associated with a Warm Core Ring positioned offshore.

**Figure 6:**
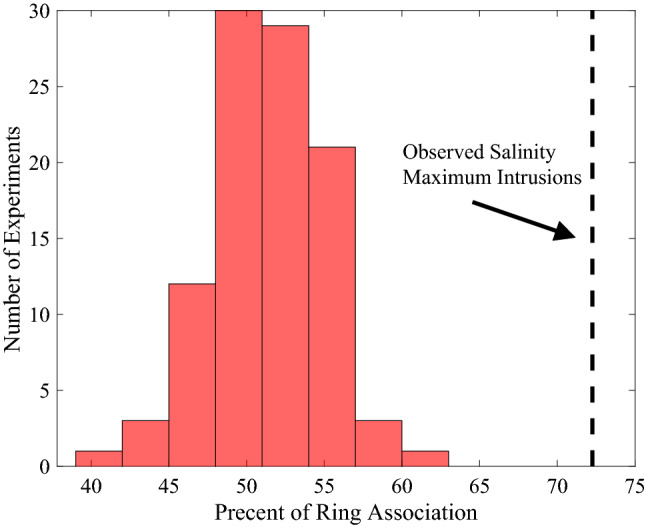
Histogram showing results from the randomized experiments. Bars show the number of random experiments with given percentages of ring association. The black dashed line shows the precent of ring association relative to the salinity maximum intrusion data.

A randomized experiment was conducted to test the reliability and validity of the above observational statistics of association. To do this, 100 experiments were conducted in which 1000 points randomly selected along the 100 m isobath were generated. For each point a random date between 1990 and 2019 was assigned and a box of ring influence was generated following the same procedure described above. In Fig. 6 the 72.3% of observed Salinity Maximum Intrusions associated with Warm Core Rings is much higher than what one would expect by chance, indicating that this is a significant relationship.

Although in the minority, some Salinity Maximum Intrusions are observed with no apparent association to a Warm Core Ring. Some of these could be generated from direct interactions between the Gulf Stream and the shelfbreak, especially to the southeast of Georges Bank where the Gulf Stream has larger meanders and has been seen to be moving further north in recent years (Fig. 4). Additionally, shingles formed off of the Gulf Stream could also generate intrusions. In recent years there have been noted changes within the Gulf Stream system. When we examine the number of Salinity Maximum Intrusions that are not associated with Warm Core Rings (a ring was not present in the box of influence within the prior month) we find that there is not a regime shift in 2000 but there does appear to be a linear trend (Figure S3). This could be due to the increasing influence of the Gulf Stream. In addition, when looking at the Taylor-Stephens Index^19^ (data available from http://www.pml-gulfstream.org.uk), an index of the Gulf Stream North Wall position, there does appear to be a linear trend, but no significant regime shift was found around the year 2000.

## Discussion

**Figure 7:**
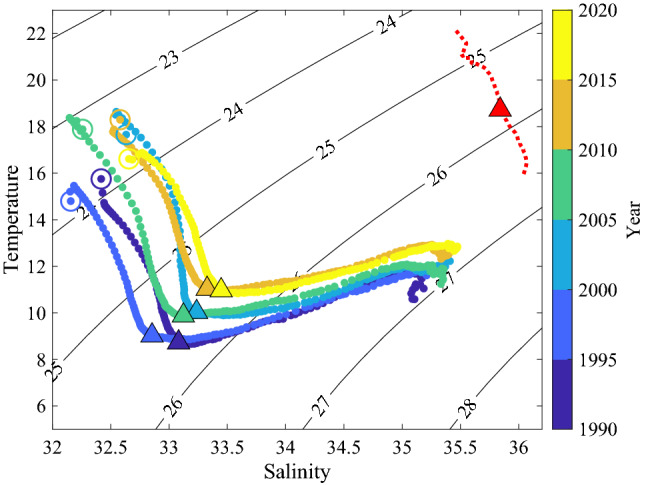
Temperature and salinity in the upper 150 m of the water column averaged over the entire shelf and in 5-year intervals from EcoMon data. The open circles demarcate the surface and the triangles mark the 50 m depth. The red dotted line is the average ring signature up to 150 m taken from Argo float data over the period 2011–2020 taken from Silver et al.^13^. The red triangle marks 50 m within the ring water. Contours of density are also shown at an interval of 1 kg/m^3^.

This work shows how changes in the offshore Gulf Stream can be indirectly linked to observed changes on the shelf mediated by Warm Core Rings forcing intrusions across the shelf break. Examining the overall average shelf water temperature and salinity we find that in recent years the shelf has become warmer and saltier, moving toward the signature of Warm Core Rings (Fig. 7). This trend is particularly evident in the top 50 m, where mid depth Salinity Maximum intrusions are likely to occur.

A key question is “How does this increase in warm salty water moving across the shelf impact the shelf ecosystem?” Several studies^6–8^ have reported biological regime shifts occurring at roughly the same time as our observed physical regime shifts. Perretti et al.^6^ described a decrease in fish recruitment around the year 2000 which they attributed to lower copepod abundance with a shift to a predominance of larger body copepods. Morse et al.^7^ also found a shift at lower trophic levels, describing a regime shift in fall zooplankton community composition and abundance in the Mid-Atlantic Bight around the year 2000. Both studies cited Greene et al.^8^ which hypothesized that the regime changes were driven by changes in atmospheric forcing, specifically changes in the phases of the North Atlantic Oscillation and Artic Oscillation, leading to changes in volume transport of Labrador Current water to the west of the Grand Banks. However after 2000, the North Atlantic Oscillation and the Arctic Oscillation both began to oscillate between negative and positive phases on much shorter timescales^8^.

These hypotheses seems to work well before 2000 and when applied to the Scotian Shelf and the Gulf of Maine; however it has been recently found that the southward influence of Labrador Current breaks down after the Great South Channel^20^, making this hypothesis harder to apply to the Southern New England Shelf and Mid-Atlantic Bight. In addition, in Morse et al.^7^ regime shifts in zooplankton populations from different ecoregions (Gulf of Maine, Georges Bank, Mid-Atlantic Bight) exhibited distinct characteristics, implying that they could have different physical drivers.

A larger number of cross slope salinity maximum intrusions could have implications for the species composition on the shelf, leading to the import of species more typically found in warmer Gulf Stream and Sargasso waters^21^. Salinity maximum intrusions have been found to extend further inshore in recent years potentially increasing the shoreward extent of these species^11^. Mid depth salinity maximum intrusions can also transport nutrients across the shelf. Friedrichs et al.^22^ found that changes in the nutrient concentration on the shelf were mostly driven by fluctuations in onshore transport. Oliver H. et al.^23^ found a subsurface diatom hotspot in the Slope Sea adjacent to the shelf which they hypothesized was generated by upwelling associated with a Gulf Stream meander. This process might also be important for the success of species whose life stages involve transport across the continental shelf such as Northern short-finned squid or the American eel^24^.

Increases in the occurrence of salinity maximum intrusions are seen alongside observed thermal changes in the shelf water. For example, data from Environmental Monitors on Lobster Traps and Large Trawlers within the Gulf of Maine (eMOLT)^25^ has revealed that bottom temperatures have been rising 0.10 °C per year from 2001 through 2021. Warming trends have also been observed in the surface shelf waters, with an increase of 0.58 °C per year observed in the upper 20 m from 2003 to 2013, greatly impacting the shelf stratification and potentially limiting nutrient availability at the surface^16^. These observed warming trends may reflect a cumulative impact of increased intrusion frequency which could ultimately compound other long-term (e.g. anthropogenic) warming trends.

In conclusion, this observational study provides a new hypothesis for the drivers of ecosystem changes observed on the Northeast US Shelf around and after the year 2000. Changes in offshore forcing by the Gulf Stream (in terms of doubling of annual formation of salty Warm Core Rings) appear to be partially responsible for observed changes in cross-shelf exchange processes in terms of quadrupling the rate of salinity maximum intrusions at mid-depth, affecting the shallow shelf waters of the Northeast US Shelf. We further assert that the recent northward movement of the Gulf Stream might also be responsible for some of the increase in the salinity maximum intrusions, both through causing intrusions directly as well as potentially forcing rings toward the shelfbreak leading to more interactions between rings and the shelf topography. As seen in Fig. 4, direct interactions between the Gulf Stream and the shelfbreak are mostly limited to the southeast of the Georges Bank region to the east of the NE shelf. Because the intrusions are subsurface features and the EcoMon dataset does not include velocity observations, it is challenging to address the dynamics of intrusion formation or evolution. Future studies using data from recent field work and numerical models will address these dynamical questions as well as the relationship between the northward position of the Gulf Stream and ring-shelfbreak interactions.

## Methods

This study uses two primary datasets. The first is the Warm Core Ring Tracking Dataset originally created by the Bedford Institute of Oceanography covering the period 1978 to 1999, and later extended through 2021^26,27^. This data set tracks weekly Warm Core Ring positions from Jenifer Clark’s Gulf Stream Charts. These charts were described and validated in multiple previous studies on ring census, operational modeling, variability and regime shifts^3,13,18,28,29^. Ring center locations and ring peripheries were digitized and quantified using a Geographic Information System QGIS framework^30^. Analysis of 10 years of Warm Core Ring tracks as well as more information on the creation of this data set can be found in Silver et al.^13^.

The other data set is a 30-year climatology of salinity maximum intrusions from Gawarkiewicz et al.^11^. This work catalogs the location, strength, depth, and frequency of salinity maximum intrusions from the Ecosystem Monitoring Program’s (EcoMon) hydrographic data. The EcoMon data covers the full extent of the shelf and is available from the 1970s, but CTD's were only gradually introduced during the 1980s. For this reason, Gawarkiewicz et al.^11^ only used data from 1990 to 2019.

In order to quantify Warm Core Ring occupancy along the shelf, an along shelf ring footprint index (RFI) was created. To create the footprint index, a probable ring impact region was first defined along the shelfbreak (black polygon in Fig. 2). This region is bounded by the 100 m isobath along its shoreward side, extending 100 km offshore of the shelfbreak. The region was sub-divided into 0.1 by 0.1 degree bins. Ring trajectories and approximate geographical range (calculated from the ring area, assuming the ring is a perfect circle) were overlain on this region and the days rings are present in each bin are counted in units of ring days. A ring day is the presence of one single ring in a bin during a given day. This methodology also accounts for the ring velocity through the Slope Sea, meaning if a ring stays in one location for an extended period, that location will have a higher ring day count. The ring area computation assumes the ring as a perfect circle, which is not entirely accurate as rings are often elliptical and a ring’s shape can change as it interacts with the shelf, with the Gulf Stream or with another ring. In addition, rings can form filaments that extend off the ring and these are not accounted for in this assumption. Despite these caveats, using this methodology we are still able to account for the relative size and respective impacts of different rings. To convert the ring occupancy value into the footprint index, the sum of all ring days from each 0.1 by 0.1 degree bin for a given month is divided by the total number of bins times the total number of days in the month. In other words, $RFI={R}_{T}/(A*T),$ where ${R}_{T}$ is the total number of ring days in the region, $A$ is the region area and $T$ is the total time. The RFI was calculated for each year the Warm Core Ring tracking data set was available (1978 to 2019) on monthly time-scales ($T$).

A number of regime-shift algorithms were applied to the different time-series presented in this study. The first is a sequential regime shift detection algorithm called Sequential Three-step Analysis of Regime Shifts or STARS^31–33^ which is freely available from https://sites.google.com/view/regime-shift-test/home. This software allows for selecting values for two important parameters that effectively control and help evaluate the magnitude and scale of the regime shift in a time-series. The first is the significance level. For all shifts detected in this work we used a significance level of 0.05 corresponding to a 95% confidence interval. The second is the cutoff length which selects for the length of the regimes. The probability of a regime being detected that is shorter than the cutoff length reduces proportional to its length. For each regime shift detected in the study we looked at a variety of cutoff lengths e.g., 5, 10, 15, 16, 17, 18, 19, and 20 years. The STARS software also has two methods of adjusting the significance level to account for red noise by adjusting for the effective degrees of freedom. These methods include ordinary least squares as suggested by Marriott & Pope^34^ and Kendall^35^ (MPK), and Inverse Proportionality with 4 corrections (IP4), both of which are described in Rodionov^33^. In addition to using the STARS software we also used the function *findchangpts* in MATLAB^36^ and compared the fit of the regime shifts versus linear models using Residual Sum of Squares (RSS). The lower the RSS, the better the fit. More details on the Regime Shift methodology and RSS can be found in Gangopadhyay et al^3^, Silver et al.^18^, and Silva^37^.

## Supplementary Information

Supplementary Information.

## Data Availability

The EcoMon data is available from the National Centers for Environmental Information World Ocean Database accessible at www.ncei.noaa.gov/products/world-ocean-database. The Warm Core Ring Tracking datasets are available from 2000–2010 and 2011- 2020 from Zenodo (https://doi.org/10.5281/zenodo.6436380, https://doi.org/10.5281/zenodo.7406675 ). The Warm Core Ring Tracking dataset from 1978 through 1999 is available from the Bedford Institute of Oceanography, Canada or from the authors upon reasonable request.
