# Supplementary material for: Increased gulf stream warm core ring formations contributes to an observed increase in salinity maximum intrusions on the Northeast Shelf
Source: Sci Rep. 2023 May 9;13:7538. doi: 10.1038/s41598-023-34494-0 (PMC10170083; doi:10.1038/s41598-023-34494-0)
Supplement: Supplementary file 1 — Supplementary Information. [file 41598_2023_34494_MOESM1_ESM.docx]

**Supporting Information**

**S1.** Figure S1 shows the interannual variability of several different metrics quantifying Warm Core Rings hitting the shelfbreak. From this figure it is clear that all four of the Warm Core Ring metrics are increasing over the 42 year period (1978-2019). There is also potentially a small shift around 2000 when the regime shift in Warm Core Ring formation occurred. A shift around this time period (ranging from 1998 to 2004) was found to be significant in all metrics using the STARS^1,2,3^ software.

**Figure S1.** Metrics of Warm Core Rings’ impact on the shelfbreak. Red lines in (a) represent cumulative metrics with the red solid line being the RFI which accounts for the time rings are present along the shelf as well as the ring’s size, and the red dotted line being the number of days rings are present along the shelfbreak each year. The blue lines in (b) are count metrics with the solid blue line being the number of rings that the hit the shelf each year and the blue dotted line being the number of rings that demised along the shelfbreak each year. Black dashed lines in both subplots mark the year 2000 when the regime shift in Warm Core Ring formation occurred.

**S2. Figure S2** shows an animation of the monthly Salinity Maximum Intrusion locations and warm Core ring Occupancy maps for two years, 1993 and 2012. These years were selected because 1993 corresponded with relatively fewer intrusions and rings and 2012 corresponded with a larger number of intrusions and rings. From this animation one can see the spatial relationship between Warm Core Rings and Salinity Maximum Intrusions, with clusters of intrusions occurring in areas adjacent to high ring occupancy. Images for each month during the 30-year period as well as the following animation are available for download on Zenodo at the following link <https://doi.org/10.5281/zenodo.7859078>^4^.


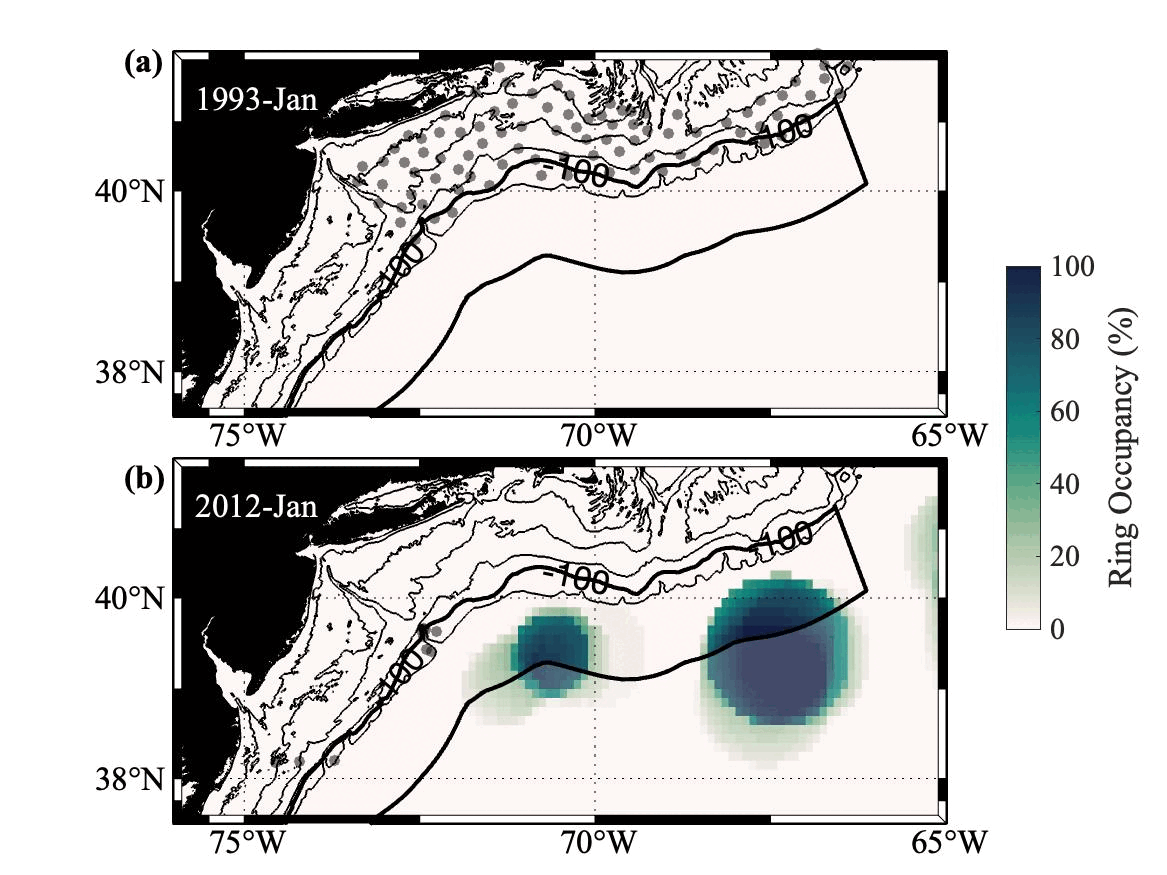


**Figure S2**. Monthly EcoMon sampling and ring occupancy for 1993 (a) and 2012 (b). EcoMon profile locations shown in red and grey dots with red dots showing profiles that contain Salinity Maximum Intrusions. The green-blue shading within the Slope Sea shows what percent of the year each region was occupied by Warm Core Rings. Thick black outline shows the region in which the ring footprint index is calculated. This region is bounded by the 100m isobath along its inshore edge and extends 1-degree offshore. The map in this animation was generated using M_Map^5^.

**S3.** Figure S3 shows the interannual variability of the Salinity Maximum Intrusion frequency for those events that are associated with Warm Core Rings (i.e. have a ring in the box of influence within the prior month) and for intrusion events that are not associated with Warm Core Rings. From this figure one can see that the intrusions that are associated with Warm Core Rings appear to have a sudden shift around the year 2000. On the other hand, intrusions that are not associated with Warm Core Rings have a more subtle shift during this time and appear to follow more of a linear trend. This linear trend in non-ring intrusions could be due to the linear trend seen in the position of the Gulf Stream North Wall (data available from <http://www.pml-gulfstream.org.uk>).

**Figure S3.** Interannual variability of Salinity Maximum Intrusion Frequency for intrusions associated (red) and not associated (blue) with Warm Core Rings.

References

1. Rodionov, S. & Overland, J.E. Application of a sequential regime shift detection method to the Bering Sea ecosystem. ICES *Journal of Marine Science* **62**, 328–332 (2005).
2. Rodionov, S.N. A sequential algorithm for testing climate regime shifts. *Geophysical Research Letters* **31**, L09204 (2004).
3. Rodionov, S.N. Use of prewhitening in climate regime shift detection. *Geophysical Research Letters* **33**, L12707 (2006).
4. Silver, A., Gangopadhyay, A., Gawarkiewicz, G. & Fratantoni, P. Monthly maps of warm core ring occupancy and occurrences of salinity maximum intrusions in the Slope Sea (1990-2019) (1.0.0). Zenodo. <https://doi.org/10.5281/zenodo.7859078> (2023).
5. Pawlowicz, R. [Computer software] M_Map: A mapping package for MATLAB, version 1.4m, available online at [www.eoas.ubc.ca/~rich/map.html](https://www.eoas.ubc.ca/~rich/map.html) (2020).
